# Tribo-biological deposits on the articulating surfaces of metal-on-polyethylene total hip implants retrieved from patients

**DOI:** 10.1038/srep28376

**Published:** 2016-06-27

**Authors:** Zhiwei Cui, Yi-Xing Tian, Wen Yue, Lei Yang, Qunyang Li

**Affiliations:** 1 School of Engineering and Technology, China University of Geosciences (Beijing), Beijing 100083, China; 2 Department of Orthopaedic Surgery and Institute of Orthopaedics, The First Affiliated Hospital, Soochow University, Suzhou, Jiangsu 215006, China; 3 AML and CNMM, Department of Engineering Mechanics, Tsinghua University, Beijing 100084, China; 4 State Key Laboratory of Tribology, Tsinghua University, Beijing 100084,China

## Abstract

Artificial total hip arthroplasty (THA) is one of the most effective orthopaedic surgeries that has been used for decades. However, wear of the articulating surfaces is one of the key failure causes limiting the lifetime of total hip implant. In this paper, Fourier transform infrared spectroscopy (FTIR), X-ray photoelectron spectroscopy (XPS) and atomic force microscopy (AFM) were employed to explore the composition and formation mechanism of the tribo-layer on the articulating surfaces of metal-on-polyethylene (MoPE) implants retrieved from patients. Results showed that, in contrast to conventional understanding, the attached tribo-layer contained not only denatured proteins but also a fraction of polymer particles. The formation of the tribo-layer was believed to relate to lubrication regime, which was supposed to be largely affected by the nature of the ultra-high-molecule-weight-polyethylene (UHMWPE). Wear and formation of tribo-layer could be minimized in elasto-hydrodynamic lubrication (EHL) regime when the UHMWPE was less stiff and have a morphology containing micro-pits; whereas the wear was more severe and tribo-layer formed in boundary lubrication. Our results and analyses suggest that enhancing interface lubrication may be more effective on reducing wear than increasing the hardness of material. This finding may shed light on the design strategy of artificial hip joints.

Since its first introduction in the 1930s, artificial hip arthroplasty has become one of the most effective procedures in orthopaedic surgery nowadays^1^,^2^. In early 1960s, Charnly designed prosthesis composed of metal femoral head and polyethylene acetabular cup, suggesting that the friction coefficient of the articulating interface between the head and the cup should be reduced to prolong the lifetime of the implant^3^,^4^. Since then, metal, polyethylene and ceramics have been used for constructing articulating surfaces. Annually, millions of patients are accepting this surgery to relieve pains and improve the performance of their impaired hip joints and this number is still increasing without any sign of slowing down. Despite the success, some problems still exist in the operation of total hip arthroplasty, such as osteolysis and prosthesis loosening. For artificial hip joints, wear of the sliding interface was believed to be the key factor limiting the life-time of prosthesis^5^. In the past decades years, the material used in the artificial hip joint was mainly metal. Majority of the researchers have focused on metal-on-metal frictional interface^6^,^7^,^8^,^9^,^10^. In 2011, Liao *et al*. found that a graphitic tribo-layer appeared on the metal-on-metal (MoM) hip replacement, which greatly helped reduce friction and wear^11^. Due to the prevalent use of metal-on-polyethylene (MoPE) implant, the similar study is also of great importance and necessity. Some studies have indicated that attached tribo-layer might not form on the sliding interface of some MoPE prosthesis^12^. While the other studies interpreted that the protein surrounding the articular cartilage might become denatured and form an attached tribo-layer on the interface after a long process of squeezing or shearing *in vivo*^8^,^12^,^13^,^14^,^15^. Therefore, over the past few years, plenty of studies have been carried out to find the correlation between the protein and the surface friction of prosthesis^1^,^6^,^7^,^8^,^11^,^13^,^16^,^17^,^18^,^19^,^20^,^21^. Apparently, it is confused that what is the key factor that influence the formation of the tribo-layer. Meanwhile, the composition of the tribo-layer is still unknown.

Fortunately, we have got three samples retrieved from patients. In sharp contrast, one sample was attached by a tribo-layer, while the others has nothing deposited on. Taking this into account, we divided the samples into two groups. In this work, the sliding surfaces of three pairs of retrieved MoPE hip implants were systematically characterized. For one of the pairs that with a harder and smoother polymer cup, was found an attached tribo-layer on the surfaces of both femoral heads and polymer cups. Nevertheless, the other pairs of sample that were more compliant and with some micro-pits on the polymer cup did not exhibit any tribo-layer attachments. By examining the difference among these polymer cups, we analyzed the composition of the tribo-layer and proposed a mechanism on how the attached film was formed and discussed the implications of the characteristics of the polymer cups to the formation of the tribo-layer.

## Results and Discussion

### Physical characteristics of materials

**Figure 1:**
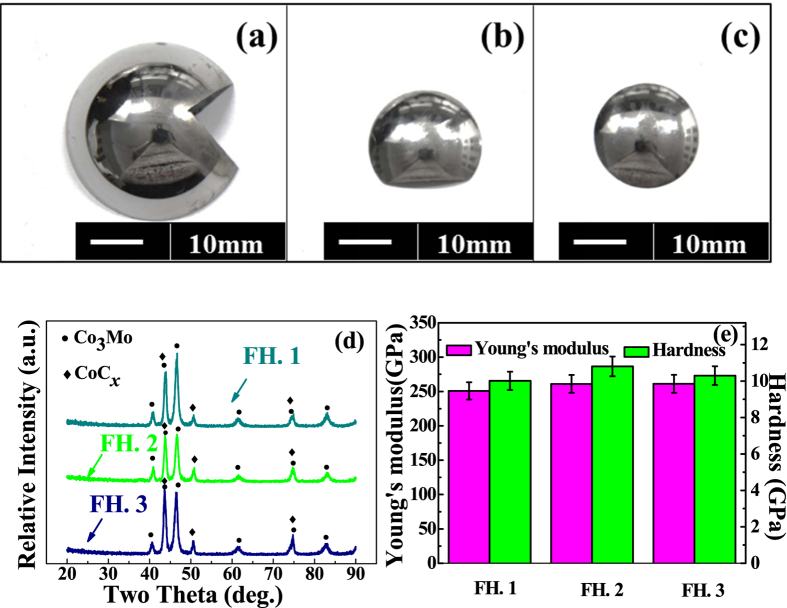
(**a–c**) represent the three pairs of prosthesis: Femoral heads were sliced by wire-electrode cutting. For simple, the No. 1 femoral head was referred to as FH. 1, the No. 2 femoral head was referred to as FH. 2 and the No. 3 femoral head was referred to as FH. 3. (**d**) was the corresponding The X-ray diffraction (XRD) results all of the femoral heads consist by the same substances and obviously the main ingredients are Co_3_Mo and CoC_x,_ (**e**) was the results of hardness and Young’s modulus of the three femoral heads.

In this work, the three pairs of samples were studied. The No. 1 sample had been used for 6 years *in vivo* and retrieved due to the failure of sliding interface. The No. 2 sample had been used for about 22 years *in vivo* and it was retrieved because of the failure occurred at the junction of the hip joint although the sliding interface of the femoral head and polymer cup still worked well. The No. 3 sample had been used for 15 years *in vivo* and retrieved for prosthesis loosening. For better characterization, the contact and sliding regions around the top of the femoral heads were cut by wire-electrode cutting and the final samples are shown in Fig. 1(a–c). Meanwhile, the corresponding physical properties were given by Fig. 1(d,e). As confirmed by the X-ray diffraction (XRD) data shown in Fig. 1(d), all the three femoral heads are made from cobalt-chromium-molybdenum alloy. The hardness and Young’s modulus measured by nano-indentation, shown in Fig. 1(e), also suggested that there were no obvious differences between the three femoral heads.

**Figure 2:**
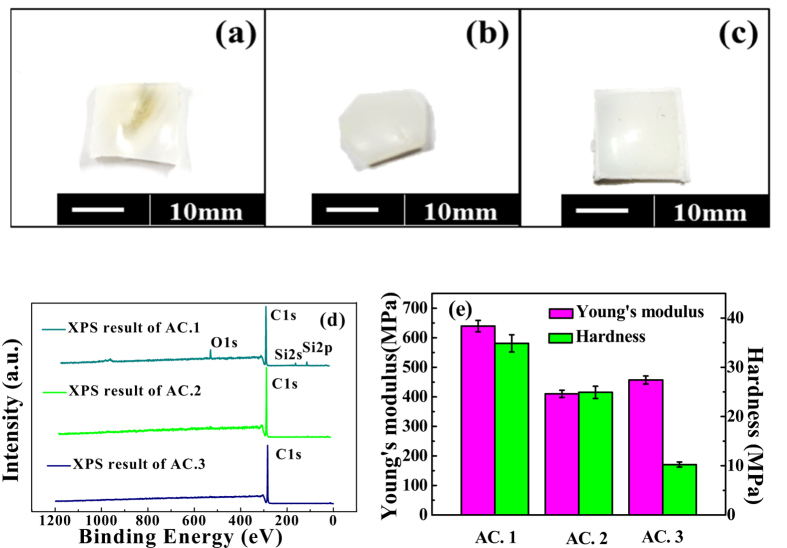
(**a–c**) were the corresponding polymer cups of the three femoral heads. (**d**) XPS results of AC. 1, AC. 2 and AC. 3, (**e**) the results of hardness and Young’s modulus of the three acetabular cups.

At the same time, the relative characteristics of each polymer cup was given by Fig. 2. It can be seen that there is a slight difference in the color of polymer cups, which suggests that the manufacturing processes of these ultra-high-molecular-weight-polyethylene are different in some extent^22^. The regions of the polymer cups were chosen and cut out by knife around the bottom, where the polymer slid against the tops of the corresponding femoral heads. From the XPS data of polymer cups shown in Fig. 2(d), one could see that there is a significant difference in their composition between the three cups. For the acetabular cup of No. 1, hereafter refers to as AC. 1, silicon was found, which maybe indicate that the AC. 1 was likely chemically cross-linked by silane^10^. While for the acetabular cup of No. 2 and No. 3, hereafter refers to as AC. 2 and AC. 3, rarely other elements can be seen in the spectrum except for carbon, which suggested that the polyethylene was likely physically cross-linked via radiation^10^, maybe γ irradiation crosslinking. The noticeable differences in the hardness and the Young’s modulus between the three polyethylene cups, shown in Fig. 2(d), also indirectly confirm the above hypothesis.

**Figure 3:**
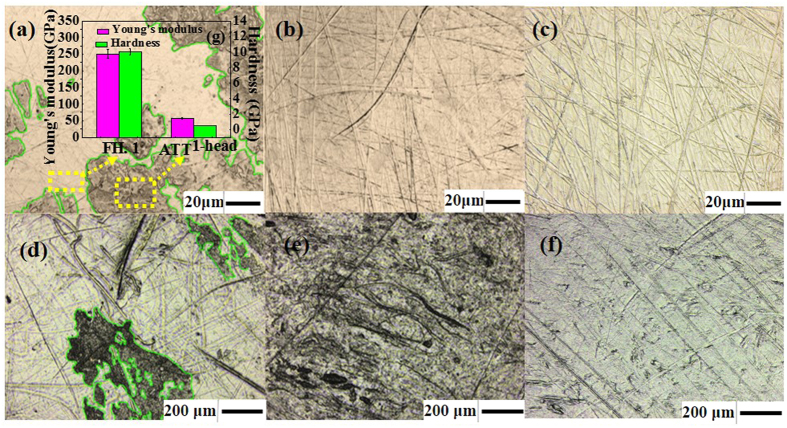
(**a–c**) represent the optical microscopy images of the femoral heads separately: (**a**) refers to the No. 1 femoral head, (**b**) refers to the No. 2 femoral head, (**c**) refers to the No. 3 femoral head, (**d**) represents the corresponding cup of No. 1, namely AC. 1, (**e**) represents the corresponding cup of No. 2, namely AC. 2. (**f**) represents the corresponding cup of No. 3, namely AC. 3 (**g**) was the results of Hardness and Young’s modulus of FH. 1and the attachment on the No. 1 femoral head, namely ATT^1-head^.

To research the difference of the tribology behavior among the prosthesis, firstly, the optical images of the samples are shown in Fig. 3. Tribo-layers were clearly found as attachments on both the femoral head and the cup of Sample No. 1, given by Fig. 3(a,d). For simplicity, the attachment on the No. 1 femoral head will be referred to as ATT^1-head^ and that on the corresponding polymer cup will be referred to as ATT^1-cup^ in the following discussion. We found that the attachments strongly adhered to the surfaces as they remained intact after ultrasonic cleaning in acetone, methanol, ethanol and deionized water in sequence for 20 mins. In sharp contrast to Sample No. 1, no attachment were discovered in sample No. 2 and No. 3 as shown in Fig. 3(b,c,e,f) instead the femoral head had slightly deeper scratches as confirmed by later inspection.

To better understand the physical properties of the attached films, we performed nano-indentation on the ATT^1-head^. As shown in Fig. 3(g), the attachment is much more compliant compared to the base material of the femoral head, *i.e.* cobalt-chromium-molybdenum alloy. Meanwhile, the atomic force microscopy (AFM) experiment was carried out on the surfaces of the femoral heads for the three samples. The topographic image of ATT^1-head^ shows that the thickness of the attached film was around 200 nm (seen in Supplementary Fig. S1(d)) and its surface friction is slightly larger than that of metal substrate (seen in Supplementary Fig. S1(c)). The high friction state of ATT^1-head^ suggested that the tribo-layer was unlikely to be graphite unlike the case of metal-on-metal contact proposed by Liao *et al*.^11^. Consistent with the optical measurement, AFM topographic images also reveal that the surface of the femoral head of sample No. 1 is smoother with shallower scratches (~10 nm) compared with the other two samples, while for the femoral head of Sample No. 2 and No. 3 the scratches are much deeper(~50 nm).

### Composition of the attachment

**Figure 4:**
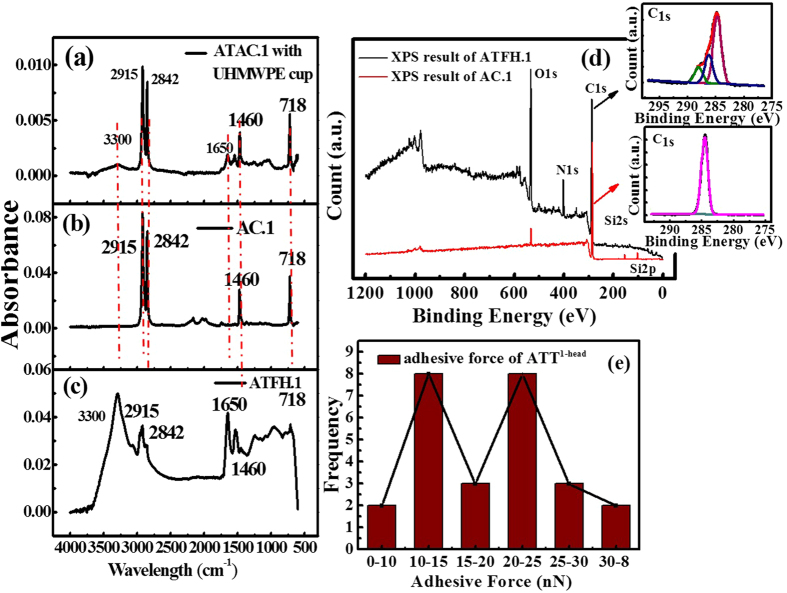
(**a**) FTIR spectra of ATT^1-cup^ with polymer cup, (**b**) FTIR spectra of AC. 1, (**c**)FTIR results of ATT^1-head^, (**d**) X-ray Photoelectron Spectroscopy (XPS) spectra of ATT1-head and AC. 1 (**e**) Adhesive force of ATT^1-head^, which was tested for 26 times.

To identify the chemical content of the attached films, the tribo-layer on femoral head and polymer cup of No. 1 sample were probed by Fourier transform infrared spectroscopy (FTIR). In order to analyze the nature of the tribo-layer, the FTIR data shown in Fig. 4(a–c).The band, as shown in Fig. 4(c), around 3300 cm^−1^ indicates the stretching vibration of N-H bonds. While, the peak appeared at 1650 cm^−1^ confirms the stretching vibration of C=O bond (coupled to the in-phase bending of N-H bond and the stretching of C-N bond)^23^. The FTIR spectra of the attachments clearly exhibit the existence of amines. The spectrum of ATT^1-cup^ is shown in Fig. 4(a). Because the thickness of the film was very small (~200 nm), the FTIR result was affected by the substrate, *i.e.* UHMWPE. The peaks arise at around 3300 cm^−1^ and 1650 cm^−1^ are consistent with that in Fig. 4(c), the FTIR result of ATT^1-head^. It can be interpreted that the tribo-layer adsorbed on the femoral head and polymer cup of No. 1 are the same in essence. The peaks at around 2915 cm^−1^, 2842 cm^−1^ and 1460 cm^−1^ represent the asymmetrical stretching vibration, symmetrical stretching vibration and bending vibration of -CH_2_-, respectively. While the peak around 718 cm^−1^ was the fingerprint peak of polyethylene indicating that the ATT^1-head^ had the characteristic of polyethylene. Connor Myant and Philippa Cann^8^ that the tribo-layer on the explanted head was composed of protein-based materials with significant γ-globulin content for metal joint through post-test FTIR Reflection-Absorption Spectroscopy, while *In vitro* simulation experiment by Mishina *et al*. hypothesized that some polymer particle might also be absorbed on metal surface for MoPE through long time rubbing^12^. It seems to us that, on sample No. 1, the essence of the tribo-layer was indeed denatured protein, meanwhile, polymer debris seemed to be indeed adhered to the surface of the femoral head as well.

For further confirm that the composition of the tribo-layer, we also performed X-ray Photoelectron Spectroscopy (XPS) to analyze the chemical state of the attached film. XPS spectra of ATT^1-head^ and AC. 1 are shown in Fig. 4(d). The main compositions of AC. 1 are C, Si and O, indicating that AC. 1 is some type of silane cross-linked polymer^10^. The detailed spectra of C1s detected on the AC and ATT^head^ were compared. It clearly shows that the bending energy of 284.6 eV responding to C=C bond in polyethylene were found for both surfaces. Considering the previous FTIR spectra, we can confirm that the attached tribo-layer formed on FH.1have the same chemical bonds with polyethylene, implying that the attached films are composed of two main components (the denatured protein and polyethylene).

We also used AFM to measure the adhesion property of the deposited film. The adhesive force of protein is less than that of the substrate referenced to Supplementary Fig. S5 and Fig. 4(e). For this, we randomly selected 26 points on the attached film and the pull-off force representing the strength of adhesion at these random locations was measured and then the histogram was plotted. According to Supplementary Fig. S5, Supplementary Fig. S6 and Supplementary Fig. S7, it is clear that the adhesive has only one peak. However, as shown in Fig. 4(e), their population of the pull-off forces on ATT^1-head^ concentrates around two distinct values, suggesting that the surface might consist of two distinct materials, which could be a further evidence.

### Formation mechanism of attachment

Since the materials of the femoral heads were nearly identical among three samples, we attributed the drastically different behavior of the sliding interface to the difference in the polymer cups of each sample. According to Fig. 3(d,e,f), the optical images of polymer cups of these samples. It can be seen in Fig. 3 that the surface of AC. 1 was overall abraded and polished with periodic scratches and a tribo-layer after long-time sliding, while the surface of the other two ACs were relatively rough with lots of surface corrugation and locally worn without any attachments.

**Figure 5:**
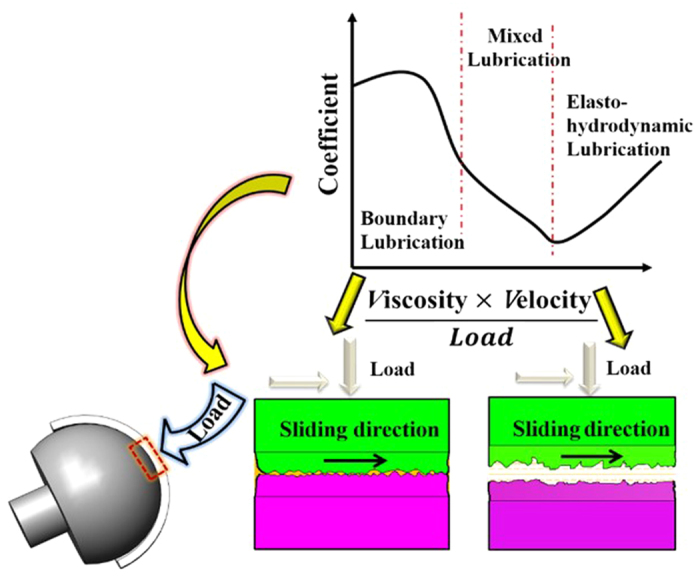
The schematic of boundary lubrication and elasto-hydrodynamic lubrication.

Based on the observations, we proposed the following mechanism for the formation of attachments as schematically shown in Fig. 5. As previously reported^11^, there are two different lubrication mechanisms for sliding interfaces of artificial hip joints: boundary lubrication^24^ and elasto-hydrodynamic lubrication^25^ (EHL).Similarly, while implanting *in vivo*, the artificial joint may work either in the regime of EHL, where a load-bearing liquid lubrication film is formed along the interface^26^, or in the regime of boundary lubrication condition, where the solid surface may contact with each other^11^,^27^,^28^. To determine whether the sliding interface is in the state of EHL, the minimum thickness of the lubrication film should be known in advance. According to Dowson-Higginson equation^26^, it can be expressed as following


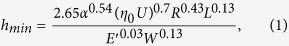


where *α* represents the viscosity-pressure coefficient, *η*_*0*_ represents the viscosity, *U* represents the average speed, *R* refers to the equivalent radius, *L* refers to the equivalent length, *E*′ represents the equivalent Young’s modulus and *W* represents the load.

We proposed that the key difference between the two groups of artificial hip joints was the value of equivalent Young’s modulus. According to the nanoindentation result of the polymer cups, it is obviously that the equivalent Young’s moduli of No. 2 and No. 3 are smaller than that of No. 1. Therefore, the thickness of the film for No. 2 and No. 3 would be larger than that of No. 1, leading to a larger separation of the sliding interfaces. In addition, from Fig. 3(e,f), lots of micro-pits appeared on the surface of AC. 2 and AC. 3, which was helpful to store lubrication fluid. From our analysis, when the artificial hip joint was squeezed, the fluid stored in the micro-pits would flow out to keep the sliding surface from contacting with each other, which is known to be beneficial for keeping the interface in the EHL lubrication state^29^,^30^,^31^,^32^. Therefore, the sliding surfaces of femoral head and polymer cup of No. 2 and No. 3 sample were better separated by the lubricant film, leading to a minor scratches and wear^26^. For AC. 1, it may be in the boundary lubrication state for a relatively long period of time, since the lubrication film cannot separate the sliding surface effectively. And from Fig. (d) the morphology of AC. 1 exhibited typical characteristics of abrasive wear which maybe confirm our suspect that it fell into boundary lubrication when a instant high contact pressure appeared. And the sliding surface of the acetabular cup will be polished, which will generate some polymer particles as third debris. Afterwards the fluid which contained various proteins will pack the debris up. After long time, the attachment perhaps will form on the two surfaces. As discussed above, when designing a pair of artificial hip joint, one can increase the hardness of the materials to improve wear resistance, while it would be more important to take the lubrication mechanism into account.

However, the authors realize the deficiency of the performed study was that all samples were retrieved from patients, after implantation history over 10 years and it is impossible to test the samples before implantation. Therefore, a portion of our conclusion were reached form speculating according to previous reports and the characteristics of the samples we got. Consequently, from our point of view, it is valuable to carry out the *in vitro* experiment to simulate the environment *in vivo* to confirm the results, which was also what we want to do next.

## Conclusion

In conclusion, we found that, under certain conditions, tribo-layer could be formed *in vivo* along the sliding interface for metal-on-polyethylene hip joints. The tribo-layer appearing on both femoral head and acetabular cup mainly consisted of denatured proteins and worn polyethylene particles. We hypothesize that the occurrence of the attached organic layer may depend on the lubrication regime. If the tribo-pairs fall in the boundary lubrication regime, it will result in major solid-solid direct contact leading to abrasive wear. The trapped debris particles will be packed up by the surrounding liquid. This in turn results in the formation of tribo-layer when the proteins are sheared mechanically at high loads and get denatured. However, if the tribo-pairs is under EHL regime, it can be achieved with minimum wear and no attachments deposited on. Considering the overall performance of the samples, our analysis suggests that enhancing interface lubrication can be more effective than purely increasing the hardness of materials when designing the artificial hip joint. A full consideration from both physiological and tribological environments inside human body is necessary to understand the wear and subsequent failure of artificial hip implants.

## Methods

### Ethic statement

The study was approved by the First Affiliated Hospital of Soochow University, Suzhou, China according to the ethical protocol (Documentation number (2012) Ethics Approval #302). The methods were carried out in accordance with the Ethical Conduct Guide of the First Affiliated Hospital of Soochow University. All patients gave written informed consent prior to participating in this study.

### Materials

All the MoPE total hip implants were retrieved from female patients after service times of 6, 22 and 15 years, respectively. There were three samples in total and they were numbered sample 1, sample 2 and sample 3 (simply No. 1, No. 2 and No. 3) separately. Sample 1 was used *in vivo* for 6 years and retrieved for the failure of contact surface *i.e* the frictional surface of the metal and polymer while sample 2 was used for 22 years and retrieved for the accidental fracture of the junction (the contact surface was still well). And sample 3 was used for 15 years *in vivo* and retrieved for prosthesis loosening. All the samples were composed of one metal femoral head and a polyethylene acetabular cup. These metallic heads were made of cobalt-chromium-molybdenum alloy while the polymer cups were made of ultra-high molecular weight polyethylene (UHMWPE). After the implants were retrieved, they were sequentially cleaned by acetone, methanol, ethanol, and deionized water to remove blood or some remaining tissues. In addition, for AFM measurements, the femoral heads had to be cut into small pieces via wire-electrode cutting.

### X-ray diffraction experiment

The X-ray diffraction (XRD) patterns were evaluated by an automated D/max-2000 diffractometer to study the phase structure of the femoral heads. The scanning range was from 20° to 90° and the scanning rate was 4°/min.

### Fourier Transform Infrared Spectroscopy experiment

The spectra of metal femoral head were gauged by a reflection infrared microspectrography, type of HYPERION which was made by BRUKER; while the spectra of polyethylene cups were gauged by an attenuated total reflection spectroscopy, type of VERTEX 70 which was made by BRUKER. During the FTIR tests the range of wave number was from 600 cm^−1^ to 4000 cm^−1^, resolution was 4 cm^−1^, acquisition time was 6 min, and the ATR was Diamond ATR annex.

### X-ray photoelectron spectroscopy experiment

As for XPS experiment, the equipment employed was PHI Quantera SXM, made by PHI. The following relative parameters were adopted in the process of this experiment: the size of beam spot was 100 μm, X-ray excitation source was Al, fine spectral flux was 55 eV and the step was 0.1 eV, full spectral flux was 280 eV and the step was 1 eV.

### Atomic Force Microscopy experiment

The AFM used here was NTEGRA PRIMA by NT-MDT. The probe employed was CSC37 Silicon tip from Mikcro Masch. All the experiments were tested at the same environment (T = 25.6 °C, RH = 39.3%). In the process of AFM experiment, the scanning rate was 1 Hz, and the scanning rang was 50 × 50 μm.

## Additional Information

**How to cite this article**: Cui, Z. *et al*. Tribo-biological deposits on the articulating surfaces of metal-on-polyethylene total hip implants retrieved from patients. *Sci. Rep.* **6**, 28376; doi: 10.1038/srep28376 (2016).

## Supplementary Material

Supplementary Information
