# Supplementary material for: Tribo-biological deposits on the articulating surfaces of metal-on-polyethylene total hip implants retrieved from patients
Source: Sci Rep. 2016 Jun 27;6:28376. doi: 10.1038/srep28376 (PMC4921821; doi:10.1038/srep28376)
Supplement: Supplementary Information [file srep28376-s1.doc]

**Supplementary information:**

**Tribo-biological deposits on the articulating surfaces of metal-on-polyethylene total hip implants retrieved from patients**

Zhiwei Cui^1^, Yi-Xing Tian^2^, Wen Yue^1*^, Lei Yang^2*^, Qunyang Li^3, 4*^

1. School of Engineering and Technology, China University of Geosciences (Beijing), Beijing 100083, China;
2. Department of Orthopaedic Surgery and Institute of Orthopaedics, The First Affiliated Hospital, Soochow University, Suzhou, Jiangsu 215006, China
3. AML and CNMM, Department of Engineering Mechanics, Tsinghua University, Beijing 100084, China
4. State Key Laboratory of Tribology, Tsinghua University, Beijing 100084,China

*Corresponding author W. Y.: Tel.: +086-10-82320255; Fax: +086-10-82322624;

E-mail address: cugbyw@163.com; [yw@cugb.edu.cn](mailto:yw@cugb.edu.cn)

* Corresponding author L.Y.: Tel: +86-512-67781540, Fax: +86-512-67781165

Email address: [leiy@suda.edu.cn](mailto:leiy@suda.edu.cn),

*Corresponding author Q.L.: Tel.: +086-10-62772933

E-mail address: [qunyang@tsinghua.edu.cn](mailto:qunyang@tsinghua.edu.cn)

**(μm)**


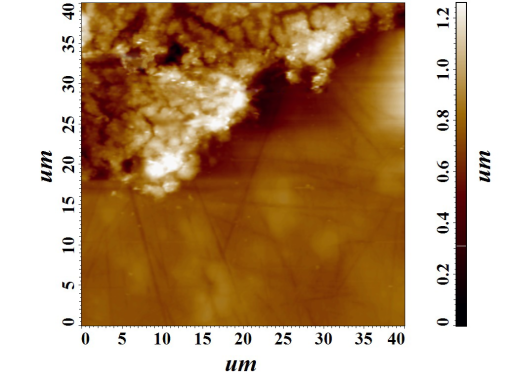

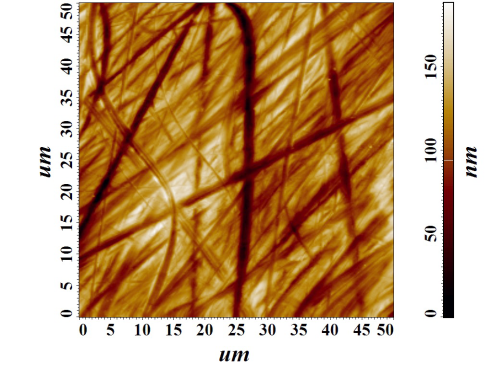


**attchment**

**substrate**

**scratch**

**(a)**

**(b)**

**(c)**

**(d)**

**(μm)**

**(μm)**

**(μm)**

**0.05**

**0.1**

**0.15**

**0**

Supplementary Figure S1 (a)AFM image of FH.1, (b)AFM image of FH.2, (c)Frictional force for FH.1, the red line represents the friction of FH.1, it is the 156^th^ line of LF image which correspond with (a), the left part is on attachment, namely it is the friction force of ATT^1-head^,and the right part is on substrate of FH.1, (d)The height of attachment


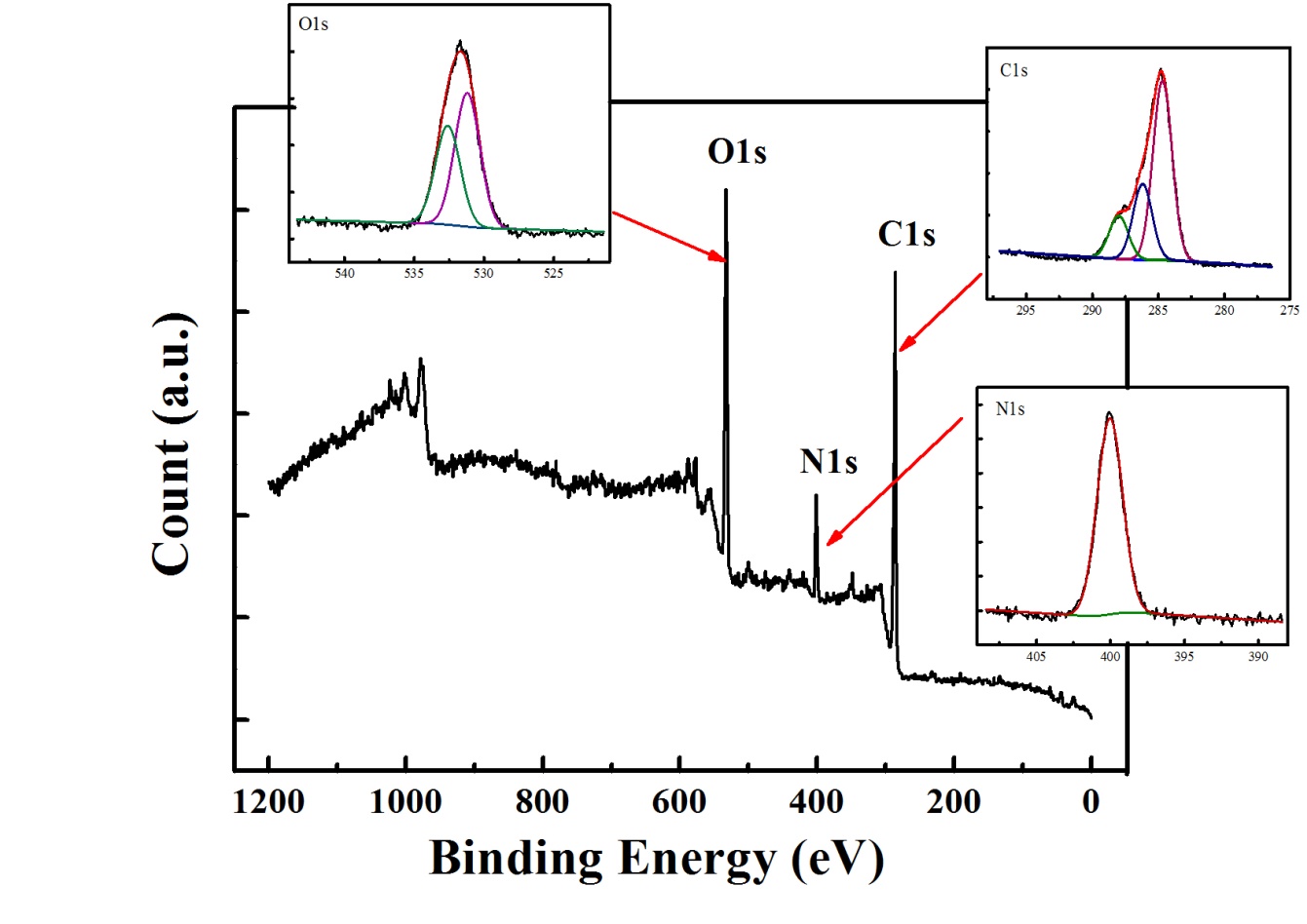


Supplementary Figure S2 XPS result of ATFH.1.


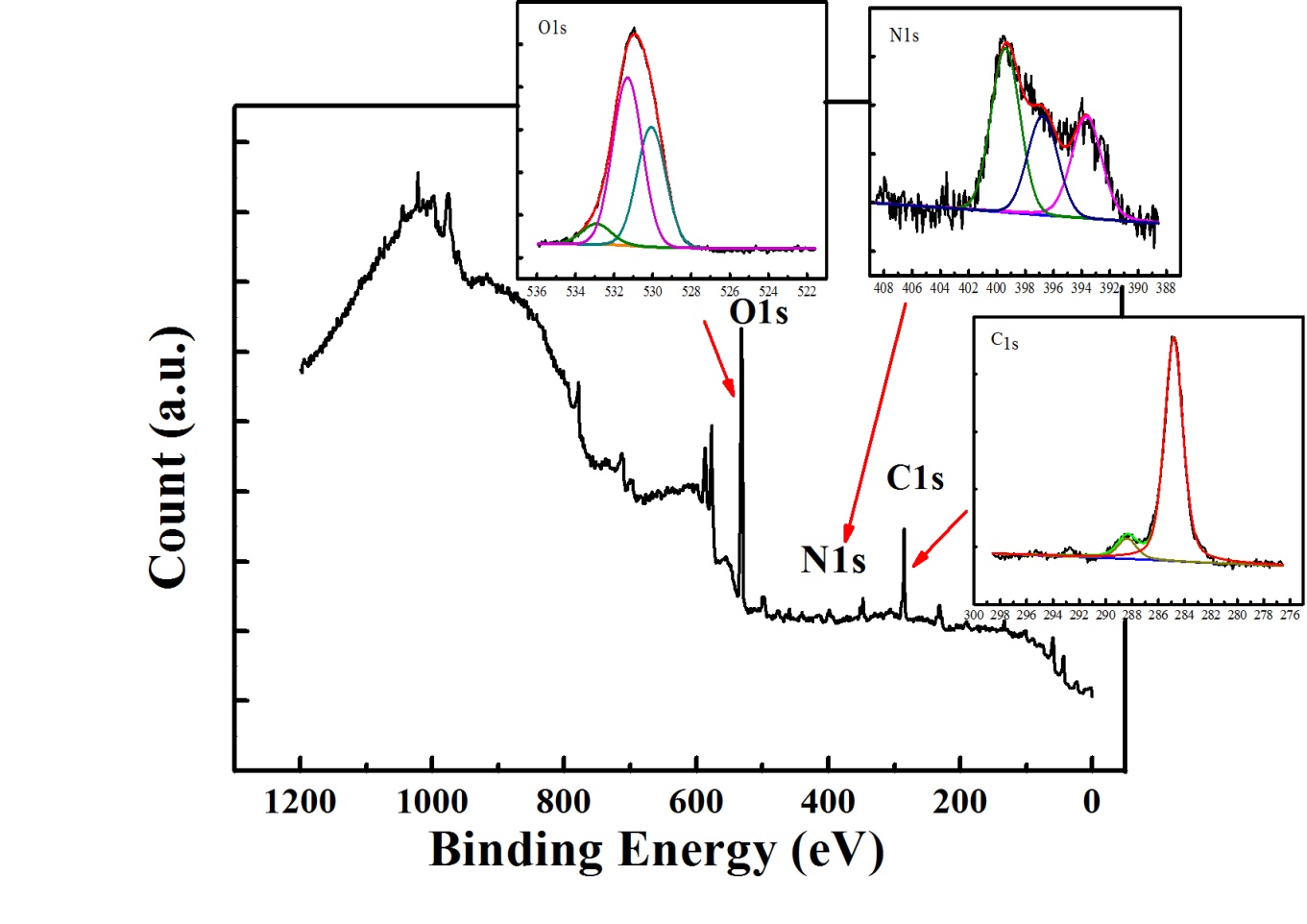


Supplementary Figure S3 XPS result of substrate for FH.1.


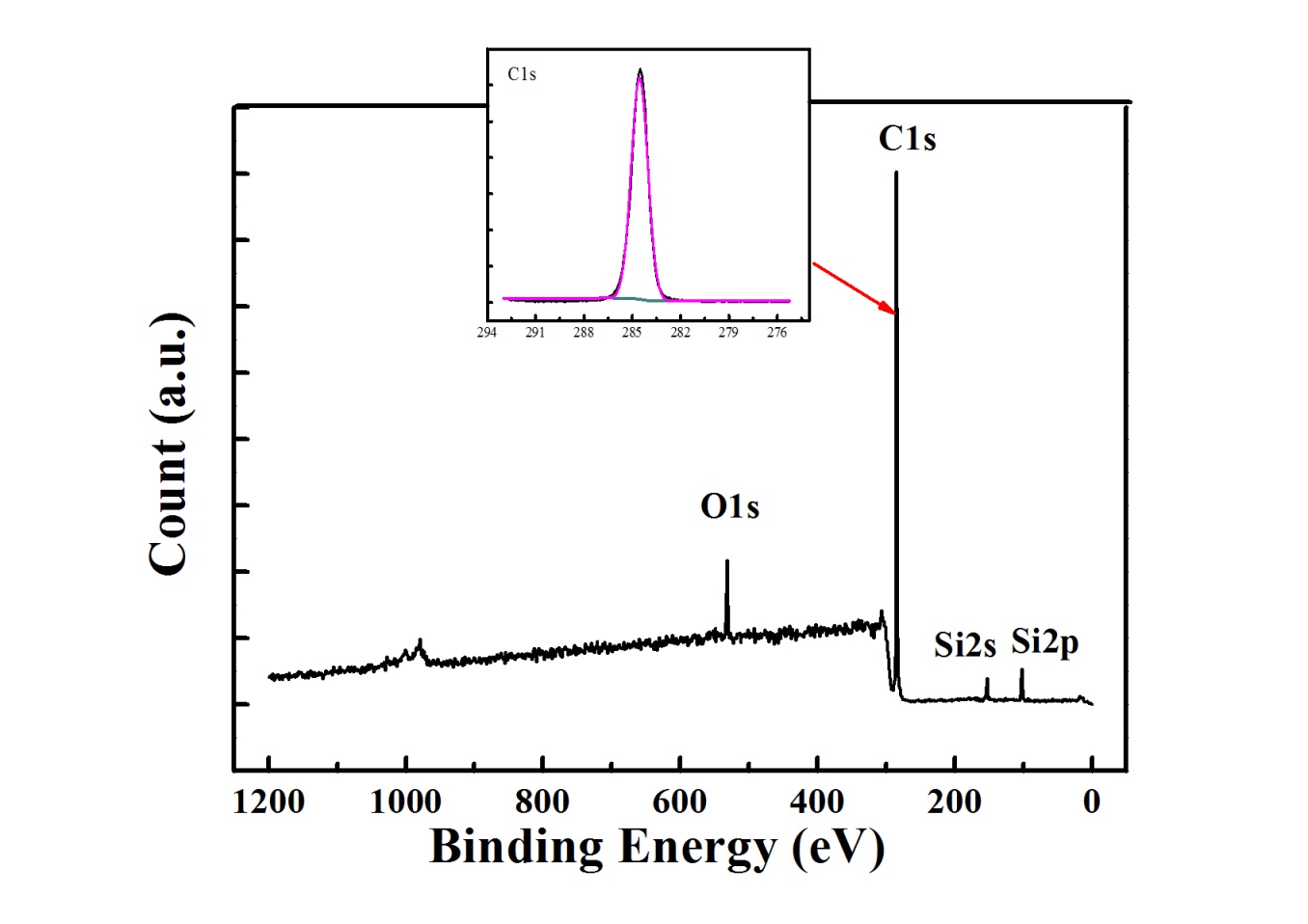


Supplementary Figure S4 XPS result of AC.1.

Supplementary Table S1 XPS result of C N O on ATFH.1(left) and substrate of FH.1 (right)

|  | **Attachment** | | | | **Substrate** | | | |
| --- | --- | --- | --- | --- | --- | --- | --- | --- |
|  | BE  (eV) | FWHM  (eV) | Area  (%) | Species | BE  (eV) | FWHM (eV) | Area  (%) | Species |
| **C 1s** | 284.6  286.2  288.0 | 1.7  1.7  1.7 | 60  25  14 | C-C C-H C=C  C-OH  C=O C-N | 284.8  288.4 | 1.65  1.7 | 91  9 | C-H  CO3- |
| **N 1s** | 400.4 | 1.8 | 100 | Organic N | 393.7  396.7  399.4 | 2.5  2.5  2.4 | 30  27  43 | Cr-N  R-NH_2_ |
| **O 1s** | 531.2  532.6 | 2.13  2.11 | 58  42 | C=O  C-OH | 530.1  531.3  532.9 | 1.76  1.8  1.8 | 38  54  7 | Cr_2_O_3_  CaCO_3_  H-O |

Supplementary Table S2 XPS result of C on AC.1

|  | **BE**  **(eV)** | FWHM  (eV) | Area  (%) | Species |
| --- | --- | --- | --- | --- |
| **C1s** | 284.6 | 1.2 | 100 | C=C |

The spectra of Supplementary Figure S2 declared the XPS results of the attachment adsorbed on No.1 femoral head. And the specific results were shown in the left part in Table 1. The spectra of Supplementary Figure S3 declared the XPS results of the substrate of FH.1. The specific results were shown in the right part in Supplementary Table S1. It is clear that the attachment consist of organics such as denatured protein while the composition of substrate is inorganic substance


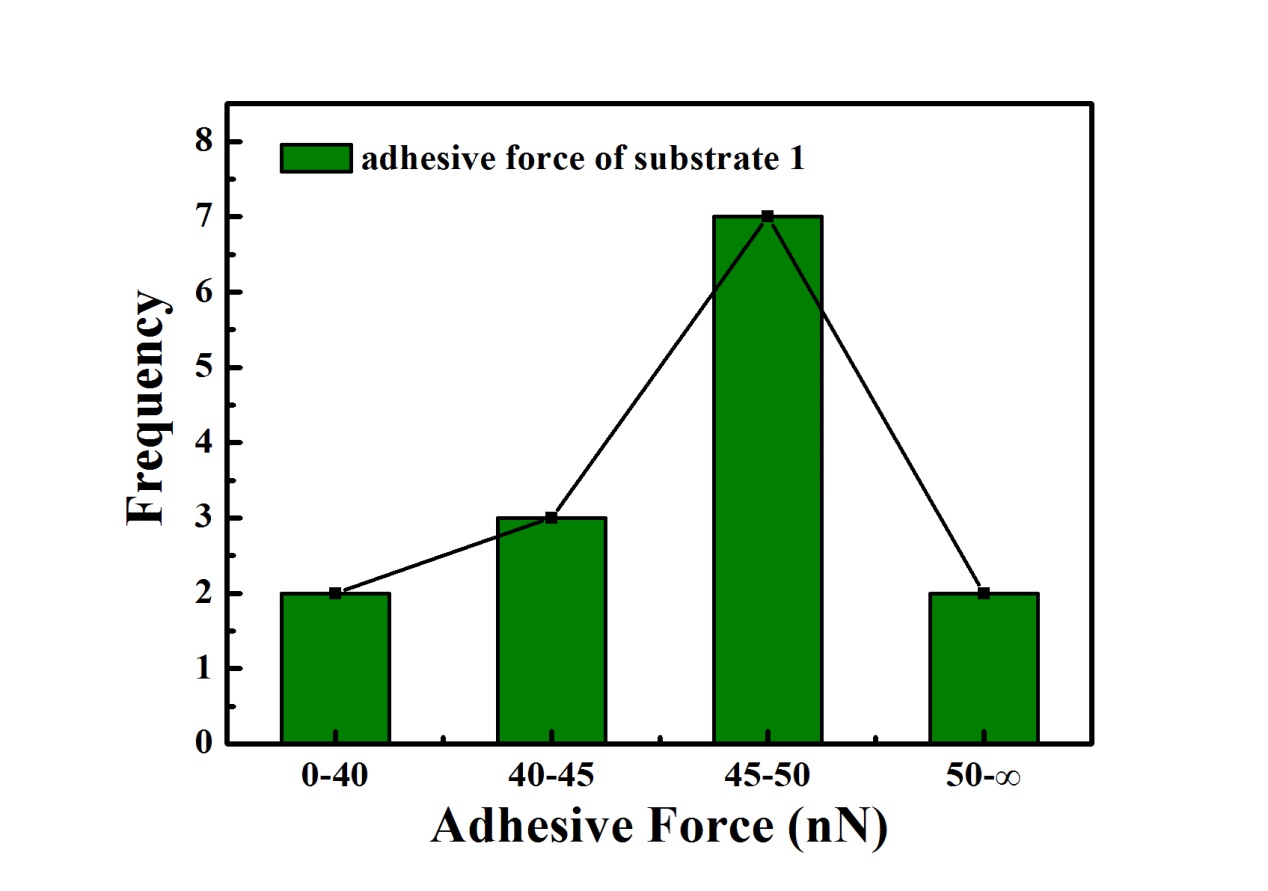


Supplementary Figure S5 Adhesive force of substrate of FH.1,which was tested for 14 times.


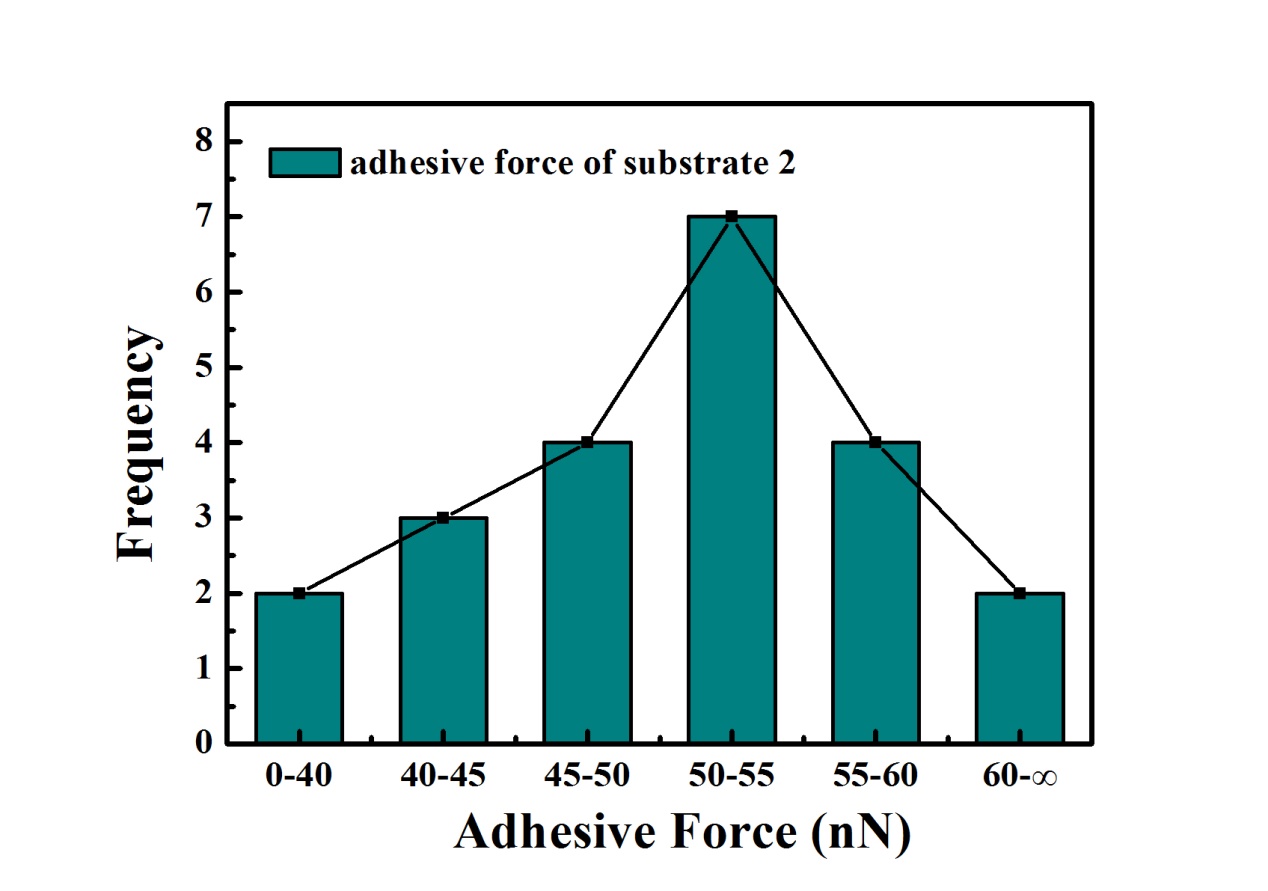


Supplementary Figure S6 Adhesive force of substrate of FH.2, which was tested for 22 times.

Supplementary Figure S7 Adhesive force of substrate of FH.3, which was tested for 14 times.
